# Nurses' attitudes towards diabetes in tertiary care: A cross‐sectional study

**DOI:** 10.1002/nop2.334

**Published:** 2019-07-23

**Authors:** Ali Hassan Alhaiti, Mohammed Senitan, Selvanaayagam Shanmuganathan, Cliff Dacosta, Linda Katherine Jones, George Binh Lenon

**Affiliations:** ^1^ School of Health and Biomedical Sciences RMIT University Melbourne Victoria Australia; ^2^ Nursing Department King Fahad Medical City Riyadh Saudi Arabia; ^3^ Department of Public Health, College of Health Sciences Saudi Electronic University Riyadh Saudi Arabia; ^4^ Menzies Centre for Health Policy, Sydney School of Public Health University of Sydney Sydney New South Wales Australia

**Keywords:** attitude, diabetes, diabetes attitude scale, healthcare providers, nurses, nursing practice, tertiary care, type 2 diabetes

## Abstract

**Aim:**

To evaluate the attitude and training of nurses in Saudi Arabia towards the care of patients with diabetes.

**Design:**

A cross‐sectional study.

**Methods:**

Data were collected in 2016 from 1,695 participants at the King Fahad Medical City using the Diabetes Attitude Scale version 3.

**Results:**

Most nurses had not received diabetes training. The agreement score of nurses for the requirement of special training for the management of diabetes is high; the survey found that most nurses were aware of the psychological effect of diabetes. However, the low agreement regarding the perception of the seriousness of diabetes among nurses and the valuing of self‐care attributes such as tight control of glycaemic level in patients with diabetes indicates the need for diabetes training.

## 1 INTRODUCTION

Given the continued increase in the number of T2D cases in all parts of the world and the urgency of the issue, there is need for increased attention by healthcare professionals, especially nurses, to offer the necessary care to patients suffering from the disease. Clark (2008) has argued that improvements in the quality of care from nurses can help in the reduction in mortality from the disease (Clark, 2008). It can also ensure that the associated complications are reduced, and patients can live a comfortable life for longer.

Healthcare services are provided by the country's Ministry of Health (MOH) at three levels: primary, secondary and tertiary care. Health systems with strong health care from primary to tertiary care are more likely to give greater attention to the management of diabetes, including specialized diabetes care nurses who share the responsibilities in managing the patients with the physicians'(Jabbour, Yamout, Giacaman, Khawaja, & Nuwayhid, 2012; Renders et al., 2001). In certain cases where individuals may be suffering from an advanced stage of disease or illness, hospitals at the tertiary level have the capability of providing treatment with the use of state‐of‐the‐art technology. Primary care centres refer patients to general hospitals, and they refer patients to tertiary hospitals for further treatment. The overall healthcare service sector in Kingdom of Saudi Arabia (KSA) is consolidated from the bottom (primary care) to the top (tertiary care; Jabbour et al., 2012).

KSA's healthcare system is growing at a rate of 2% per year to satisfy the rising demand for healthcare services brought about by increased growth of the population as well as a surge in chronic diseases (Jabbour et al., 2012). Currently, patients with diabetes are managed at all three levels of health care by endocrinologists, internists and general practitioners. Since the care for patients with diabetes involves several medical disciplines including nephrology, cardiology and ophthalmology, specialized diabetes centres and clinics are necessary to work as liaising bodies.

For most countries in the world, the quality of care by nurses and other healthcare professionals, especially in the public health sector, has been poor. According to Fitzgerald et al. (2008), this is due to the attitudes of healthcare professionals towards patients with diabetes and the attitudes of patients towards service providers (Fitzgerald et al., 2008). This has resulted in a reduction in the rates of adherence to self‐care management activities that patients should practice daily and a reduction in the essential daily self‐monitoring of blood glucose.

Given that nurses in the healthcare setting spend the most time with patients, they are better positioned to give education and offer more care to patients with T2D than other healthcare professionals. In addition, nurses are usually better positioned than other healthcare professionals, such as doctors and physicians, to recommend to patients the best care practices and various measures to manage the disease. Lou et al. (2014) argued that nurses are better listeners and have better knowledge of patients with diabetes than other healthcare professionals. This means their attitude and commitment to the care of patients with T2D should be higher than that of other healthcare professionals. Nurses' attitude is important in influencing patients care and management of their diseases.

Research shows that nurses possess the lowest attitude and perception about care of patients with T2D. For instance, nurses' decisions to improve patient management and the care they offer are motivated by perceptions of seriousness of the disease. Nurses' attitudes play an important role for specialized care for certain diseases (Blaser & Berset, 2019). If they perceive a disease to be less serious, they will offer lower quality care and will be less concerned about offering care services. Given that most nurses have encountered diseases they consider deadlier than T2D, there is a poorer perception and attitude among nurses of the seriousness of T2D. This means that, with respect to care for people with T2D, nurses have a poorer attitude to offering good services; greater effort and concern is directed to patients suffering – from the nurse's point of view – from more serious diseases.

Offering quality care to patients with T2D requires specialized training to impart the knowledge of the necessary day‐to‐day self‐care management and monitoring activities. This also helps with gaining knowledge about various lifestyle changes patients with diabetes should make to minimize associated disease complications. According to Odili and Oparah (2012), nurses with specialized training in the care and monitoring of patients with T2D have a better attitude and perception towards their patients than nurses who have not received such training (Odili & Oparah, 2012). This is because they have a better understanding of the needs of such patients and good knowledge of the care activities they require. Hence, nurses' perceptions and attitudes towards care for patients with T2D are greatly influenced by whether they have received specialized T2D training.

Another aspect that affects nurses' attitudes about the care of patients with T2D is their attitude towards daily blood glucose management. One of the most important factors for patients with T2D is to monitor their blood glucose level daily; this can greatly influence their health because T2D results from high blood sugar levels compared with insulin levels in the blood. For nurses who value the need to control blood glucose levels daily, their perceptions and attitudes towards the care of patients with T2D are high. This is the opposite for nurses who do not see the value of controlling blood glucose levels on a daily basis. Nurses who value control of blood glucose levels understand the need to offer such care to patients and have no issues with doing so (Bisheya, El‐Mijbri, Beshyah, & Sherif, 2011). Those who do not appreciate the need to control blood glucose levels daily may not wish to give such care services to patients with diabetes and, hence, their poorer attitude and perception.

The perception of nurses of the effects of the disease on patients is another factor that influences the perception and attitude of nurses about care for patients with T2D. If they perceive the effects on patients to be substantial, they offer high‐quality services and hold positive attitudes towards offering care and monitoring patients on a daily basis. This is because the seriousness of a disease affects the level of care and the attitude nurses hold towards offering care to patients: those with less serious diseases receive less care and attention than those with diseases perceived to be more serious.

The autonomy of patients with T2D is another factor that may affect nurses' perceptions. Most healthcare professionals, including nurses, believe that daily self‐care and self‐monitoring activities should be carried out by patients, and hence, patients should receive the necessary training and be empowered to do this (Babelgaith, Alfadly, & Bahari, 2013). This is because patients are the ones who experience the various bodily effects of the disease, and thus, they are better positioned to take any necessary measures, but only if they are well educated about what to do. The aim of this study was to evaluate the attitude and training of nurses in Saudi Arabia towards the care of patients with diabetes.

## 2 METHODOLOGY

The study was conducted in King Fahad Medical City (KFMC) in Saudi Arabia, one of the largest healthcare facilities in the Gulf Region, with a total capacity of 1,200 beds. Based on hospital data, the KFMC employs a total of 3,295 nurses in total who are able to switch between jobs in different departments, including the Specialised Diabetes and Endocrine Centre (SDEC) Diabetes Centre. The population included all nurses in KFMC, from which a sample of 1,695 participants was targeted for recruitment. The inclusion criteria for the participants were as follows: all nurses who are employed as full time, age of participants from 21–60 years of age, has 1 year of work experience and they can read and write in Arabic or English.

Data collection continued for a period of five months. It was expected that after five months the researcher would have collected enough data to meet the minimum number of samples required for the study. At the end of the data collection period, the researcher encoded the data in Excel and transferred all the data to SPSS. All statistical calculations were undertaken using the SPSS v. 23 software.

During the recruitment phase, nurses who were interested to participate in the study were given a survey package. In the survey package, an informed consent form was provided. The participants were asked to sign the informed consent form prior to responding to the DAS‐3 study instrument. Participants were allowed to respond to the survey questionnaire at their own convenient time and location. Participants were asked to submit the survey package to a box located in the Nursing Department.

To achieve the desired 95% confidence level for a 0.05 margin of error, a sample size of 1,695 was required (Odili & Oparah, 2012). However, the study managed to recruit only 1,126 participants.

Data were collected using the Diabetes Attitude Scale (DAS‐3) developed by the Michigan Diabetes Research and Training Centre (Al‐Maskari et al., 2013). Permission to use this survey questionnaire was obtained from the respective authors. The DAS‐3 can be administered to both patients and healthcare professionals to assess general attitudes towards diabetes. The DAS‐3 consists of five subscales pertaining to perceptions on diabetes and its management, namely the need for special training, seriousness of non‐insulin‐dependent diabetes mellitus, value of tight control, psychosocial effect of diabetes mellitus and patient autonomy (Al‐Maskari et al., 2013). Nurses were asked to indicate their level of agreement with the 33 statements in the instrument, based on a five‐point Likert‐type scale ranging from 1 (strongly disagree)–5 (strongly agree). This study received ethics approval from the ethics committee at KFMC in Saudi Arabia (H‐01‐R‐012), IRB with OHRP/NIH, USA (IRB00008644) and RMIT University in Australia (ASEHAPP 59–14).

## 3 RESULTS

**Figure 1:**
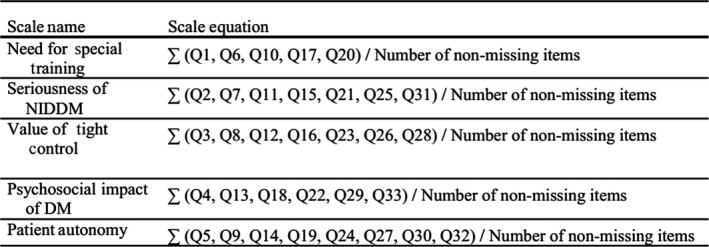
Diabetes attitude scale‐3 formulae

Scores were calculated for each domain by summing the score for all questions pertaining to that domain and dividing that score by the total number of responses (Figure 1). For example, the need for special training domain was calculated by summing scores for all participants from questions 1, 6, 10, 17 and 20 and then dividing the sum by the total of the responses for all these questions.

### 3.1 Participants' demographic data

Demographic data collected with the DAS‐3 survey included age group, occupation, gender, nationality, years of experience and diabetes training. The results presented in Table 1 show that 96.6% of the participants were nurses (*N =* 1,088), and only 3.4% were clinical educators (*N =* 38). The largest age group represented in the survey is 31–40 years (*N =* 452, 40%). Most participants were non‐Saudi (*N =* 1,075, 95.5%); only 4.5% were from the KSA (*N =* 51).

**Table 1:** Summary of participants' demographic characteristics

Characteristic	*N*	%
Occupation		
Nurse	1,088	96.6
Clinical educator	38	3.4
Age group (years)		
21–30	440	39.1
31–40	452	40.1
41–60	234	20.8
Gender		
Male	64	5.7
Female	1,062	94.3
Nationality		
Saudi	51	4.5
Non‐Saudi	1,075	95.5
Years of experience		
1	45	4.0
1–5	321	28.5
6–10	420	37.3
11–15	206	18.3
>15	134	11.9
Diabetes training		
Yes	243	21.6
No	883	78.4

More than half of the participants were female (*N =* 1,062, 94.3%), and 5.7% were male (*N =* 64). They varied in terms of experience: 1–5 years (*N =* 321, 28.5%), 6–10 years (*N =* 420, 37.3%), 11–15 years (*N =* 206, 18%) and >15 years' experience (*N =* 206, 11.9%). Most (*N =* 883, 78.4%) had not received any prior diabetes training.

### 3.2 Nurses' perception regarding care for patients with type 2 diabetes

As demonstrated in Table 2, there was high agreement among nurses that diabetes educators require special training: the mean agreement score is 4.37, which is very high and indicates strong agreement. The same is noted for the patient autonomy domain where the mean agreement score is 3.8, which indicates that the nurses were aware that patients with diabetes should participate in planning their own care plans and determining their own glycaemic goals.

**Table 2:** Perception score summary for nurses regarding diabetes care

	Mean	Standard deviation
Need special training	4.37	0.50
Seriousness of diabetes	3.03	0.49
Value of tight control	3.34	0.51
Psychosocial effect of DM	3.68	0.49
Patient autonomy	3.82	0.45

**Figure 2:**
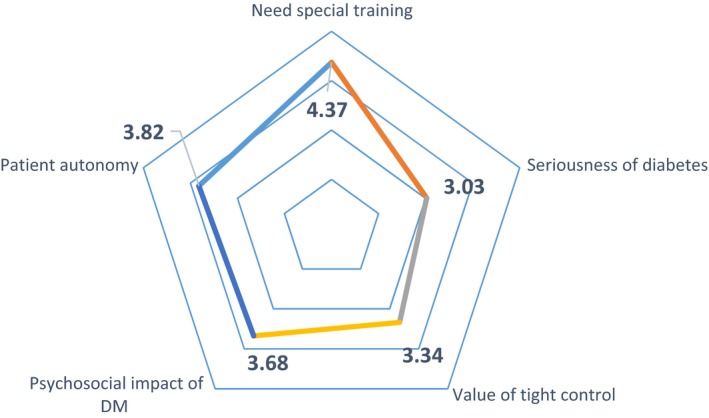
Summary of responses to DAS‐3 survey

As demonstrated in Figure 2, the mean agreement score regarding the psychological effect of DM domain is 3.68, which lies between 3 (neutral)–4 (agree), indicating a degree of agreement. This shows that nurses were aware of the psychological effect of T2D. However, the seriousness of diabetes domain has a mean value of 3.02, which indicates that nurses held differing opinions regarding the seriousness of the disease. The same can be said for the value of the tight control domain where the mean score is only 3.3. This can be explained by the fact that almost 80% of the nurses did not receive diabetes training, which may have led them to underestimate the value of tight glycaemic control and the seriousness of the disease.

## 4 DISCUSSION

The attitudes of healthcare providers (HCPs), specifically nurses, towards diabetes are important as nurses are the first point of contact for patients with diabetes. Several studies have assessed the attitudes of HCPs towards diabetes and how HCPs perceive different aspects of that disease. An examination of the literature identified many studies performed in the United States, Europe, South America and Asia to assess the attitudes of physicians, pharmacists and nurses towards diabetes, as attitudes play an important role in how HCPs communicate with patients to give health care. A few studies have been conducted in Gulf countries (such as the UAE), other Arab countries (such as Yemen) and countries in Africa (including Nigeria and South Africa) (Table 3). The current study adds new information to the existing literature about how HCPs (specifically nurses) perceive diabetes and its seriousness in the KSA.

**Table 3:** DAS‐3 subscale mean results in several countries, including the current study

	KSA	UAE	Yemen	Nigeria
Need special training	4.4	4.4	4.2	4.4
Patient autonomy	3.8	3.6	3.3	3.5
Psychosocial effect of DM	3.7	3.88	3.4	3.33
Value of tight control	3.4	3.51	3.4	2.95
Seriousness of diabetes	3.0	3.8	2.6	3.17

Most nurses included in the current study (78.4%) had not received diabetes training. The number of nurses who had received diabetes training was less than that reported in the UAE, where more than half of the nurses included in the study by Bani‐Issa, Eldeirawi, and Al Tawil (2014) had received diabetes training, compared with only 24.6% in our study (10). Further, 36.0% of the UAE nurses had received diabetes certification. The educational level of nurses is an important factor that affects how HCPs perceive diseases such as T2D. Bani‐Issa et al. (2014) used DAS‐3 scores to assess the attitudes of HCPs in their study, as was done here. Regarding the need for special training, the average score in this study is similar to that reported by Bani‐Issa et al. (2014) (*M* = 4.4). This score was the highest in both studies: Bani‐Issa et al. (2014) found that the highest mean attitude score was on the need for special training (4.5 in HCP and 4.4 in nurses). This indicates that nurses in both the KSA and other Gulf countries (such as the UAE) understand their own shortfalls in handling problems associated with diabetes and its management.

Comparing other scales of the DAS‐3 between the two studies, several differences were found. The score for patient autonomy in the current study is higher than that reported by Bani‐Issa et al. (2014) (3.8 vs. 3.6) (10). This indicates that nurses in the KSA understand that patients have the right to participate in the formulation of their care plan and have a positive attitude towards patient autonomy, which ranked second with respect to score (*M* = 3.82). This finding suggests that nurses believe that patient with diabetes should participate in formulating their own care plan and contribute to decisions concerned with their self‐management.

The main surprise among findings in the current study is the low attitude score for the seriousness of diabetes (mean = 3.02) compared with results from other countries. For example, the study performed in the UAE reported overall mean = 3.8 for HCPs and nurses. This indicates that the attitude towards the seriousness of diabetes is low in the current sample, which may negatively affect how nurses communicate with diabetic patients. This difference with respect to the UAE study may be explained by the fact that 50% of the nurses in the UAE study had received diabetes training and almost 18% had diabetes certification (Bani‐issa et al., 2014). These numbers are higher than the numbers reported in the current study, where only 21.6% of nurses had received diabetes training. These results indicate that diabetes training and certification may be important in the KSA to raise awareness among HCPs regarding the seriousness of patients with T2D and that continuing education programmes are necessary to achieve such outcomes.

There are further differences between the two studies. Bani‐Issa et al. (2014) included a smaller number of nurses compared with the current study, although they came from multiple centres across the UAE, unlike the current sample, which came from a single medical centre. Regarding attitudes towards the psychological effect of DM, the value of tight glycaemic control and patient autonomy, results from the current sample do not differ significantly from those reported for UAE nurses.

Comparing the current results with those from a study of Yemen by Babelgaith et al. (2013), an interesting finding is that most nurses included in the Yemeni sample were males, whereas the sample in the current study consisted mainly of females (Babelgaith et al., 2013). In addition, the final number of Yemeni nurses was very small (*N =* 17). Nonetheless, there is good agreement between the results of the two studies. For example, the need for special training ranked first in Yemen, with a score of 4.2, which concords with the results here. Moreover, attitudes towards the seriousness of diabetes and the value of tight glycaemic control are similar in the two studies. However, the attitude scores towards the psychological effect of diabetes and patient autonomy are higher in the current study.

With respect to countries in Africa, Odili and Oparah (2012) carried out similar research in Benin City in Nigeria to explore the attitudes of nurses, pharmacists and physicians towards diabetes care (Odili & Oparah, 2012). Nurses had a similar attitude towards the seriousness of diabetes (*M* = 3.17) as that reported in the current study (*M* = 3.0). Likewise, they reported that the attitude score towards the need for special training was high, similar to the current study. This further confirms that nurses are aware of their relative lack of knowledge when it comes to communicating and dealing with diabetic patients (Odili & Oparah, 2012).

Van Zyl and Rheeder (2008) examined the attitudes of medical and nursing staff at Kalafong Hospital in South Africa and found that although HCPs had average to poor knowledge about the care for diabetes, they held positive attitudes towards patients with diabetes and understood that special training in diabetes is necessary (van Zyl & Rheeder, 2008). Nonetheless, van Zyl and Rheeder (2008) confirmed that the need for special training was the highest scoring domain among the five domains assessed by the DAS−3. None of the scales scored below 3.5, which indicates high awareness regarding diabetes. Patient autonomy scored 3.75, and the mean score of the psychological effect of diabetes was 3.67.

With respect to attitudes towards the psychological aspects of diabetes, the mean attitude score in the current study is 3.69, which is lower than the score reported for Nigeria (*M* = 4.07) (Odili & Oparah, 2012) but similar to that for Yemen (*M* = 3.70) (Babelgaith et al., 2013). These results were expected, as most healthcare professionals related to diabetes focus on clinical guidelines and pathophysiological, biological and psychosocial aspects/treatments to prevent late macrovascular and microvascular complications of diabetes.

Unlike in other studies (Babelgaith et al., 2013; Gagliardino, González, & Caporale, 2007; Odili & Oparah, 2012), the autonomy subscale here is not the lowest of all the attitude subscales. This may be because most nurses in the current study had not received appropriate training regarding diabetes. Thus, seriousness of diabetes is the lowest of all domains in the current study.

Regarding the value of tight glycaemic control, the mean score in the current study is 3.33, which is slightly lower than scores in the literature (Babelgaith et al., 2013; Bani‐issa et al., 2014). As tight glycaemic control is very important to prevent diabetes complications, the attitudes of HCPs towards tight glycaemic control should be improved to ensure proper management of the disease (Mannucci, Dicembrini, Lauria, & Pozzilli, 2013).

One proposed solution to improve the attitudes of HCPs towards diabetes is through continuing education programmes. Indeed, training programmes should be implemented at regular intervals to ensure that HCPs are up to date with knowledge regarding diabetes and to improve the attitudes of HCPs towards diabetes. The government could also establish special training institutions to ensure quality provision of services to various patients. There is a difference in the care needed for other patients and for patients with T2D. As such, nurses and other medical personnel may seek to attend forums, seminars and workshops aimed at ensuring quality provision of health care to patients with diabetes. E‐Learning could be used as another option to improve nurses' knowledge and attitudes towards patient care. Nursing attitudes towards patients can be improved by acknowledging the perceived patients' beliefs on the habits, tradition and culture.

## 5 CONCLUSION

Healthcare practitioners, especially nurses, play an important role as providers of care in hospitals and as the workers in direct contact with diabetic patients. Thus, their attitudes towards diabetes and its management are important to ensure provision of optimal health care to patients with diabetes. Nurses in the current study believed strongly that there is a need for special diabetes training to care for patients with diabetes; this implies that they understood the complexity of diabetes as a disease and were aware of their inadequacies in handling the various clinical, humanistic and economic issues associated with its management. These findings accord with the published literature, as nearly all studies to have assessed attitudes towards diabetes have highlighted the importance of special training on diabetes. The MOH should promote Arabic language courses for foreigners so they can communicate more effectively with the local population.

As only 21.6% of nurses had received diabetes training in current study, diabetes training and certification may be important in the KSA to raise awareness among HCPs regarding the seriousness of patients with T2D. Further, continuing education programmes are necessary to achieve such outcomes. Nurses in the KSA understood that patients have the right to participate in the formulation of their care plan and held positive attitudes towards patient autonomy, which ranked second with respect to score (mean = 3.82). This finding suggests that nurses believe that patients with diabetes should participate in formulating their own care plan and contribute to decisions concerned with their self‐management. The patients should be properly advised regarding the importance of strict control of their glycaemic levels to prevent severe physiological effects of T2D; this can be achieved if nurses and HCPs are aware of the seriousness of T2D and are trained to teach and recognize the importance of strict glycaemic control. Proper management in terms of diagnosis and treatment is essential with involvement of policymakers in KSA government to relook at current system and should seek to invest in ensuring proper service delivery and better accessibility to programmes and institutions that can help with issues related to diabetes. The involvement of the government in the disease issue will go a long way towards improving the lives of patients with diabetes.

## 6 RELEVANCE TO CLINICAL PRACTICE

The study findings are significant towards diabetes patient care and nursing education. Nurses in KSA need special training in diabetes to tackle the increasing burden of diabetes among the population. These findings imply that greater concern should be made to address the issue by hospital administrators and nurse managers. Effective strategies are recommended for better quality and service for nursing care in KSA.

## CONFLICTS OF INTERESTS

All authors have read and understood the Nursing Outlook declaration of conflicts of interests. We declare that we have no competing interests in this project.

## AUTHORSHIP STATEMENT

All listed authors meet the authorship criteria, and all authors are in agreement with the content of the manuscript.

## DATA SHARING STATEMENT

No additional data are available for sharing.
